# Efficacy of PARP inhibitors in advanced high-grade serous ovarian cancer according to BRCA domain mutations and mutation type

**DOI:** 10.3389/fonc.2024.1412807

**Published:** 2024-09-09

**Authors:** Roberto Buonaiuto, Giuseppe Neola, Aldo Caltavituro, Alessandra Longobardi, Federica Pia Mangiacotti, Amedeo Cefaliello, Maria Rosaria Lamia, Francesco Pepe, Jole Ventriglia, Umberto Malapelle, Giancarlo Troncone, Mario Giuliano, Grazia Arpino, Sandro Pignata, Carmine De Angelis

**Affiliations:** ^1^ Scuola Superiore Meridionale (SSM), Clinical and Translational Oncology, Naples, Italy; ^2^ Department of Medical Oncology, School of Medicine and Surgery, University of Naples Federico II, Naples, Italy; ^3^ Public Health Department, University of Naples Federico II, Naples, Italy; ^4^ Urology and Gynecology Department, Istituto Nazionale Tumori IRCCS, Fondazione G. Pascale, Naples, Italy

**Keywords:** HGSOC, PARP inhibitors, BRCA mutation domain, resistance, ovarian cancer

## Abstract

**Objective:**

Preclinical studies have emphasized the potential connection between BRCA specific domains defects and the activity of Poly ADP-ribose polymerase inhibitors (PARPi). Nevertheless, real-world evidence regarding the impact of BRCA domain defects and mutations on PARPi efficacy are limited. The aim of his study was to evaluate the efficacy of PARPi in terms of progression free survival (PFS) according to BRCA domains defects and mutation types.

**Methods:**

A retrospective analysis was performed among 79 BRCA mutated patients, diagnosed with advanced High-grade serous ovarian carcinoma (HGSOC) who received first- and second-line platinum- based chemotherapy followed by PARPi maintenance treatment. PFS was evaluated according to BRCA1 [Really Interesting Gene (RING), DNA Binding (DBD), Serine Cluster (SCD), BRCA1 C-terminal (BRCT)] and BRCA2 [RAD-51 Domain (RAD-51 BD), DBD] specific domain defects and mutation types [missense (MS), nonsense (NS), frameshift (FS), splicing (S), or large rearrangements (LR)].

**Results:**

After a median follow-up of 51 months, no significant difference in PFS was observed between the BRCA functional domains or mutation types in the BRCA1 and BRCA2 subgroups. Patients with BRCA2 DBD and RAD51-BD defects had the longest (39.8 months) and shortest (24.1 months) median PFS, respectively (p = 0.11). Additionally, patients with BRCA1 DBD defects had the greatest benefit (median PFS = 33.8 months) while those with BRCA1 RING domain mutations experienced the worst outcome (median PFS = 30.9 months (p = 0.43).

**Conclusion:**

The efficacy of maintenance treatment with PARPi is independent by BRCA domain defects or mutation types. Patients DBD domain defects experienced numerically longer median PFS compared to those with other BRCA1/2 alterations.

## Introduction

Ovarian cancer is a significant contributor to global cancer-related deaths, and it is the leading cause of death for gynecological malignancies (1, 2). Although platinum-based chemotherapy combinations are the primary treatment for epithelial ovarian cancer, recent years have seen the introduction of targeted therapies, including humanized monoclonal antibody against vascular endothelial growth factor (VEGF) bevacizumab, inhibitors of poly (ADP-ribose) polymerase (PARPi), and antifolate receptor alpha antibody drug conjugates, expanding the pharmacologic landscape.

High-grade serous ovarian carcinoma (HGSOC) is the most prevalent histological subtype of epithelial ovarian cancer and is associated with mutations in the breast cancer susceptibility genes 1 and 2 (*BRCA1* and *BRCA2*) in approximately 25% of cases (3–6). BRCA1 and BRCA2 are tumor suppressor genes whose mutations have been historically associated with an increased lifetime risk of developing breast cancer (50 -80%) and ovarian cancer (30 - 50%) (7). BRCA1 and BRCA2 proteins are crucial interactors in the Homologous Recombination (HR) DNA double-strand break (DSB) repair system, which ensures genomic stability and DNA integrity during the S and G2 cell cycle phases (8, 9). BRCA1 also plays a role in other DNA repair pathways, including the error-prone non-homologous end-joining (NHEI) DSB repair system and single-strand annealing (SSA) (10). Ovarian cancer cells with mutations in *BRCA1*, *BRCA2*, or genes involved in the HR mechanism have an Homologous Recombination Deficiency (HRD) status, which renders them highly dependent on the DNA single-strand break (SSB) repair mechanism to maintain genomic stability. Notably, the DNA SSB recognition and repair process is primarily driven by chromatin - associated proteins, such as poly (ADP-ribose) polymerase, which promote the synthesis of poly (ADP- ribose) chains to recruit DNA repair factors to SSBs and ensure cell survival (11).

The inhibition of poly (ADP-ribose) polymerase (PARP) in BRCA-deficient ovarian cancer cells impairs the DNA damage response and induces cell apoptosis through a phenomenon known as “synthetic lethality.” (12). However, the range of PARP inhibition sensitivity extends from primary resistance to long-lasting responses. Interestingly, the degree of tumor sensitivity to PARP inhibition can be influenced by specific BRCA domain defects. BRCA1, for example, has several functional domains, including the amino terminal RING domain, which promotes BRCA1-BARD1 (BRCA1 Associated RING Domain protein 1) heterodimer formation and its E3 ubiquitin ligase activity (13). The BRCT domain mediates binding to phosphorylated proteins like CtBP-interacting protein (Ctlp) and ABRAXAS, promoting recruitment to DNA damage sites, DNA end resection, and G2/M checkpoint activation (14). The binding domain directly interacts with DNA regions, functioning as both a DNA damage sensor and repair promoter (15). BRCA2 has two functional domains: the RAD51-binding domain, which contains BRC repeats that bind to RAD51 and promote its recruitment to the double-strand break (DSB) to ultimately form RAD5 filaments on single-strand DNA (ssDNA), and the DNA binding domain, which is crucial for mediating BRCA2 interaction with both ssDNA and double-strand DNA (16). Preclinical studies have investigated the potential impact of BRCA-specific domain loss on treatment activity. In genetically engineered mice with two common *BRCA1* frameshift mutations (BRCA1185delAG and BRCA15382insC), the loss of the BRCA1 ring domain was associated with resistance to both cisplatin and olaparib (17). Conversely, the expression of a BRCA2 variant lacking the DNA Binding and C-terminal domains resulted in defective BRCA2 architectural rearrangement, leading to increased PARPi sensitivity in murine cell lines (18).

Limited clinical data are available on the impact of BRCA-specific defects on PARPi efficacy. A *post-hoc* analysis of the PAOLA 1 trial showed that although the benefits of the combination regimen have been observed regardless of BRCA domains, mutations affecting the BRCA2 DNA-binding domain (DBD) are associated with increased sensitivity to platinum salts treatment and improved median Progression Free Survival (mPFS). Conversely, defects in the BRCA1 DBD were correlated to a worse clinical outcome, indicating reduced sensitivity to platinum salts treatment in this patient subgroup (19). Therefore, understanding the pattern of PARPi sensitivity based on BRCA domain defects may help predict the extent of PARP inhibition’s benefits and improve the clinical management of patients with HGSOC.

## Methods

### Patients’ selection

A retrospective, multicenter study of electronic health records from patients who were referred to the oncology departments of the University Hospital Federico II, IRCCS Fondazione Pascale, and AORN Cardarelli Hospital between April 2016 and September 2023 was undertaken. The study population comprised patients aged 18 years or older with an Eastern Cooperative Oncology Group Performance Status (ECOG PS) of 0-2 who were treated with platinum-based chemotherapy as first- or second-line treatment followed by maintenance therapy with PARPi. Eligible patients had a histological diagnosis of FIGO stage III or IV high-grade serous ovarian cancer (HGSOC) with a germline or somatic *BRCA1* or *BRCA2* mutation. The study included patients who underwent primary debulking surgery (PDS) or interval debulking surgery (IDS), regardless of the surgical outcome. PARPi treatment could be administered for up to 24 months (olaparib) or 36 months (niraparib) in patients with no evidence of disease (NED) in the first- line setting. In patients with residual disease and in the second-line setting, PARPi was administered until disease progression, death, or unacceptable toxicity. All patients treated with second-line PARPi received platinum-based first-line treatment, followed by a maintenance regimen with bevacizumab.

### Genetic characteristics

Patients were included if they had tested positive for pathogenic germline or somatic mutations in *BRCA1* or *BRCA2* genes. Patients with BRCA1/2 variants of unknown significance (VUS) (n = 8) or those carrying mutations in any other genes involved in the Homologous Recombination Repair (HRR) were excluded from the analysis. The mutation records were classified based on the consultation of the two largest BRCA mutation databases, BRCA Exchange (20) and ClinVar (21), according to mutation type and BRCA protein functional domains. Pathogenic variants were classified as missense (MS), nonsense (NS), frameshift (FS), splicing (S), or large rearrangements (LR). The BRCA1 protein functional domains were defined as the RING Finger Domain (amino acids 8-96), the DNA Binding Domain (DBD, amino acids 452-1092), the Serine Cluster Domain (SCD, amino acids 1280-1524), the BRCA1 C-terminal (BRCT, amino acids 1646-1736 and 1760-1855). The BRCA2 protein functional domains were defined as the RAD51-Binding Domain (RAD-51 BD, amino acids 900-2000) and the DNA Binding Domain (DBD, amino acids 2459-3190). Any other mutation not involving those reported amino acid regions for BRCA1 or BRCA2 was classified as “Other.”

### Statistical analysis

Recorded data were processed using Jamovi (22–24) software, version 2.3.28. The follow-up time was determined from the date of diagnosis to October 2023. Progression-free survival (PFS) was measured from the date of diagnosis to the time of disease progression. If disease progression did not occur, patients were censored at the date of their last follow-up. PFS was analyzed and visualized using the Kaplan–Meier method, and the comparison was made using the log-rank test. The Cox-regression model was used to evaluate hazard ratios (HR), which were expressed in 95% confidence intervals (95% CI). A significance level of 5% (p ≤ 0.05) was considered statistically significant. The study was approved by the Research Ethics Committee, and patients provided informed consent for the use of their clinical data and tumor samples (BiOnCam protocol).

## Results

### Cohort characteristics

The baseline demographic and clinical characteristics of the 79 consecutive patients included in this study are presented in **Table 1**. The median age at diagnosis was 58 years, with a range of 34 to 79 years. Sixty- two (78%) patients were diagnosed with stage III HGSOC, while 17 (22%) had stage IV HGSOC. Of these patients, 54 (68%) underwent PDS, and 25 (32%) received IDS. Fifty-six (71%) patients received first-line platinum-based chemotherapy, followed by maintenance therapy with PARPi. Twenty-three (29%) patients received platinum-based chemotherapy as first-line treatment, followed by maintenance therapy with intravenous bevacizumab. All these patients experienced a platinum-sensitive disease recurrence or progression and received a second-line platinum-based chemotherapy followed by PARPi.

**Table 1:** Baseline patients’ characteristics.

Age	
Mean (SD)	57.8 (± 11)
Median (min-max)	58 (34-79)
ECOG Performance Status, n (%)	
0	62 (78.5)
1	12 (15.2)
2	5 (6.3)
FIGO Stage, n (%)	
III	63 (79.7)
IV	16 (20.3)
Surgery, n (%)	
Primary Debulking Surgery	54 (68.4)
Interval Debulking Surgery	25 (31.6)
PARP Inhibitors line therapy, n (%)	
1st Line	56 (70.9)
2nd Line	23 (29.1)
PARP Inhibitor administered, n (%)	
Olaparib	70 (88.6)
Niraparib	5 (6.3)
Rucaparib	4 (5.1)
Mutated Gene, n (%)	
BRCA 1	48 (60.8)
BRCA 2	31 (39.2)
BRCA 1 Protein Domain affected, n (%)	
RING Finger Domain	6 (12.5)
DNA Binding Domain	13 (27.1)
Serine Cluster Domain	6 (12.5)
BRCT Domain	15 (31.2)
Other	8 (16.7)
BRCA 2 Protein Domain affected, n (%)	
RAD51-Binding Domain	11 (35.5)
DNA Binding Domain	9 (29)
Other	11 (35.5)
Mutation Type in BRCA 1 gene, n (%)	
Missense	9 (18.8)
Frameshift	16 (33.3)
Large Rearrangement	5 (10.4)
Splicing	4 (8.3)
Nonsense	14 (29.2)
Mutation Type in BRCA 2 gene, n (%)	
Missense	3 (9.7)
Frameshift	10 (32.3)
Splicing	4 (12.8)
Nonsense	14 (45.2)
Residual tumor during PDS and IDS, n (%)	
R0	60 (75.9)
R1	8 (10.1)
R2	11 (14)
1st line chemotherapy, n (%)	
Carboplatin - Paclitaxel	65 (82.2)
Carboplatin - Paclitaxel + Bevacizumab	12 (15.2)
Carboplatin - Gemcitabine + Bevacizumab	1 (1.3)
Carboplatin – Pegylated liposomal doxorubicin	1 (1.3)
Number of cycles of 1st line chemotherapy, n (%)	
6 cycles	77 (97.4)
5 cycles	2 (2.6)
2nd cytoreductive surgery, n (%)	
Yes	2 (8.7)
No	21 (91.3)
Second cytoreductive surgery residual tumor, n (%)	
R0	1 (50)
R1	1 (50)
R2	0 (0)
Second line chemotherapy, n (%)	
Carboplatin – Paclitaxel	4 (17.4)
Carboplatin – Gemcitabine	13 (56.5)
Carboplatin – Gemcitabine – Bevacizumab	3(13.0)
Carboplatin – Pegylated liposomal doxorubicin	2 (8.7)
Carboplatin – Pegylated liposomal doxorubicin – Bevacizumab	1 (4.4)
Number of cycles of 2nd line chemotherapy, n (%)	
6 cycles	17 (73.9)
5 cycles	4 (17.4)
4 cycles	2 (8.7)
1st line PARP dose reduction, n (%)	
Yes	18 (32.1)
No	38 (67.9)
2nd line PARP dose reduction, n (%)	
Yes	12 (52.2)
No	11 (47.8)
1st line AE G3-G4, n (%)	
Anemia	7 (46,2)
Neutropenia	2 (13,2)
Thrombocytopenia	5 (33)
Increase of AST or ALT concentration	1 (6,6)
2nd line AE G3-G4, n (%)	
Anemia	6 (45,6)
Neutropenia	1 (7,6)
Thrombocytopenia	5 (38)
Increase of AST or ALT concentration	1 (7,6)
1st line treatment discontinuation, n (%)	
Yes	0 (0)
No	56 (100)
2nd line treatment discontinuation, n (%)	
Yes	1 (4,4)
No	22 (95,6)
1st line AE G3-G4, n (%)	
Anemia	7 (46,2)
Neutropenia	2 (13,2)
Thrombocytopenia	5 (33)
Increase of AST or ALT concentration	1 (6,6)
2nd line AE G3-G4, n (%)	
Anemia	6 (45,6)
Neutropenia	1 (7,6)
Thrombocytopenia	5 (38)
Increase of AST or ALT concentration	1 (7,6)
1st line treatment discontinuation, n (%)	
Yes	3 (5)
No	53 (95)
2nd line treatment discontinuation, n (%)	
Yes	1 (4,4)
No	22 (95,6)

### Distribution of mutations in BRCA1 and BRCA2

**Figure 1:**
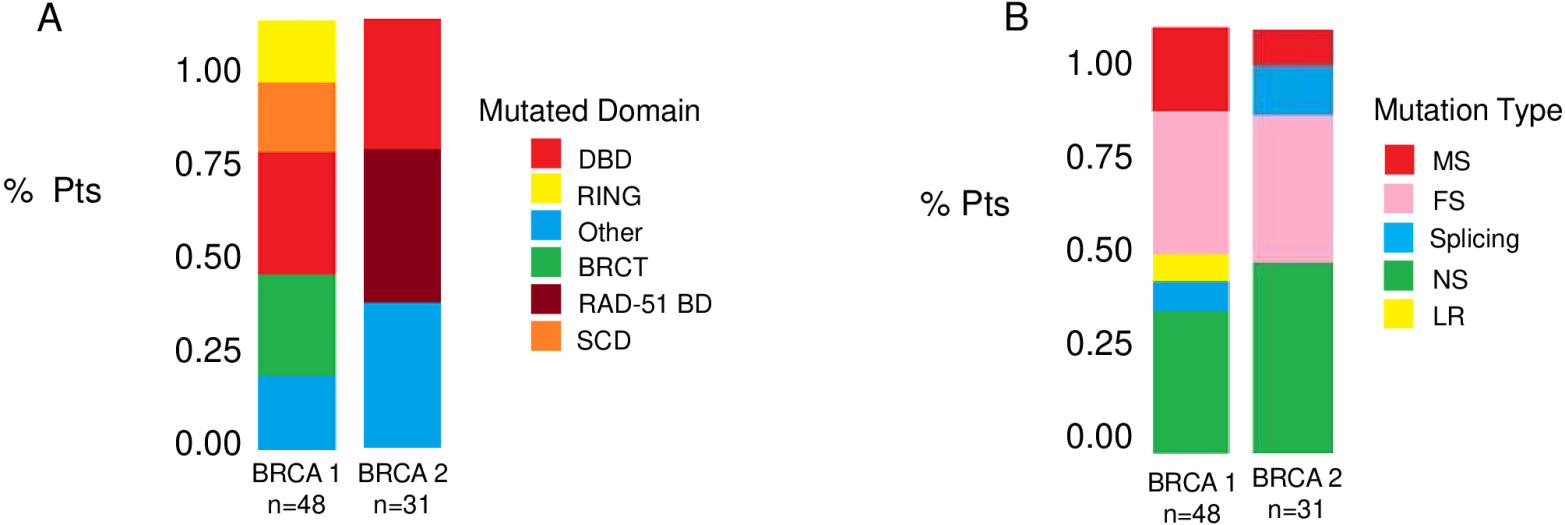
Distribution of BRCA1 and BRCA2 mutations. Bar plots show the frequencies of BRCA mutations according to BRCA specific domain defects **(A)** and to mutation type **(B)**. BRCT, C-terminal domain of BRCA1; DBD, DNA-binding domain; RING, Really Interesting New Gene; RAD51 BD, RAD51-binding domain; SCD, serine cluster domain; MS, missense; FS, frameshift; MS, missense; LR, large rearrangement.

**Figure 1** and **Table 1** summarize the BRCA1 and BRCA2 mutation type and location. *BRCA1* and *BRCA2* mutations were detected in 48 (61%) and 31 (39%) patients, respectively. The BRCT (15 cases, 31%) and the DBD (13 cases, 27%) were the most common mutated domains in BRCA1-mutated HGSOC. Among patients with BRCA2-mutated HGSOC, 11 (35%) harbored a mutation in RAD51-BD, and 9 (29%) had a mutation in the DBD. Notably, a substantial number of mutations occurred outside the identified functional domains for BRCA1 (8 cases, 17%) and BRCA2 (11 cases, 35%) (**Figure 1A**). According to mutation type, FS (16 cases, 33%) were the most common alterations for *BRCA1*, and NS (14 cases, 45%) were the most frequent for *BRCA2* (**Figure 1B**).

### Progression-free survival

**Figure 2:**
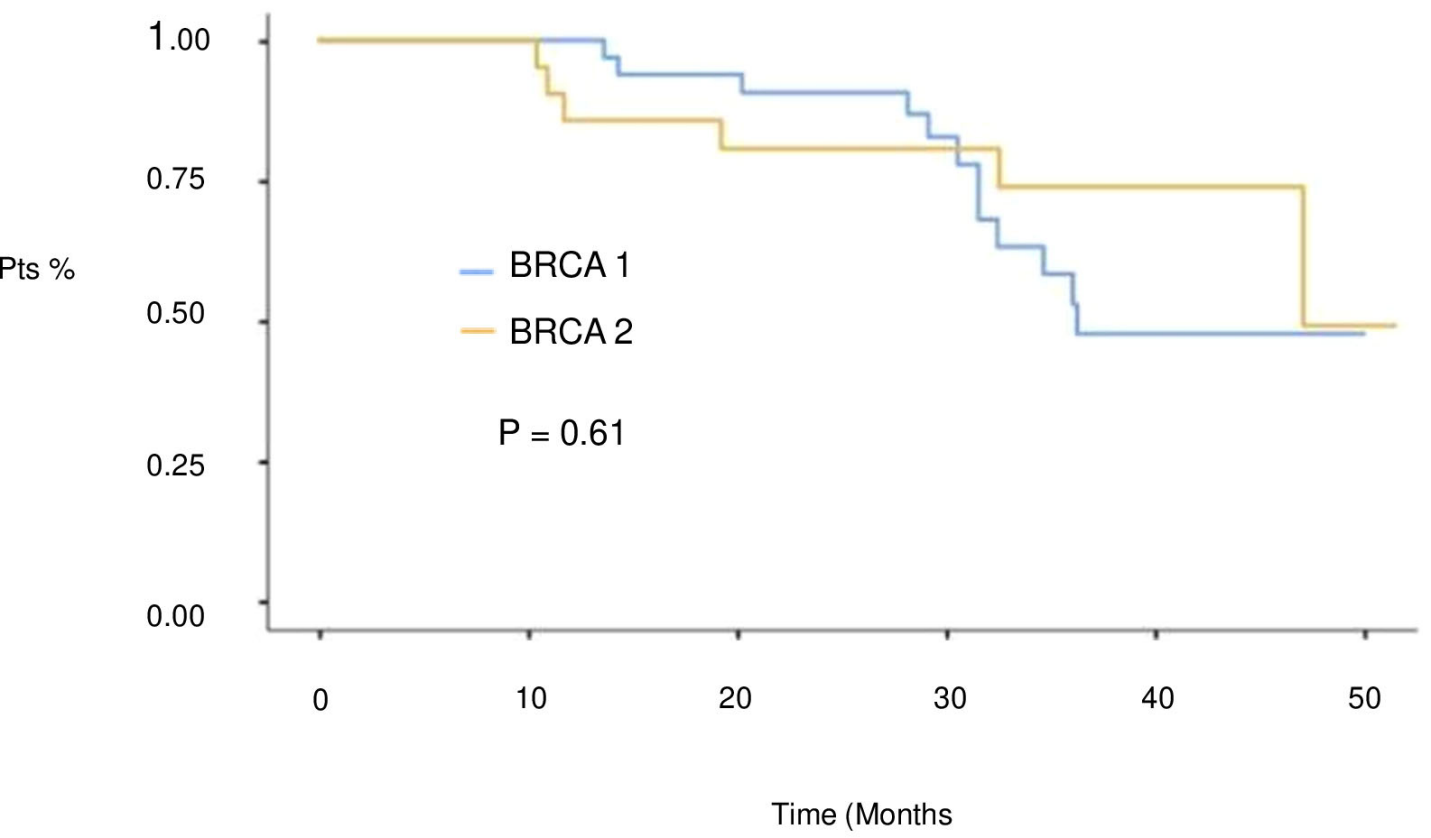
PFS in 1st line setting according to BRCA1 and BRCA 2 genes.

**Figure 3:**
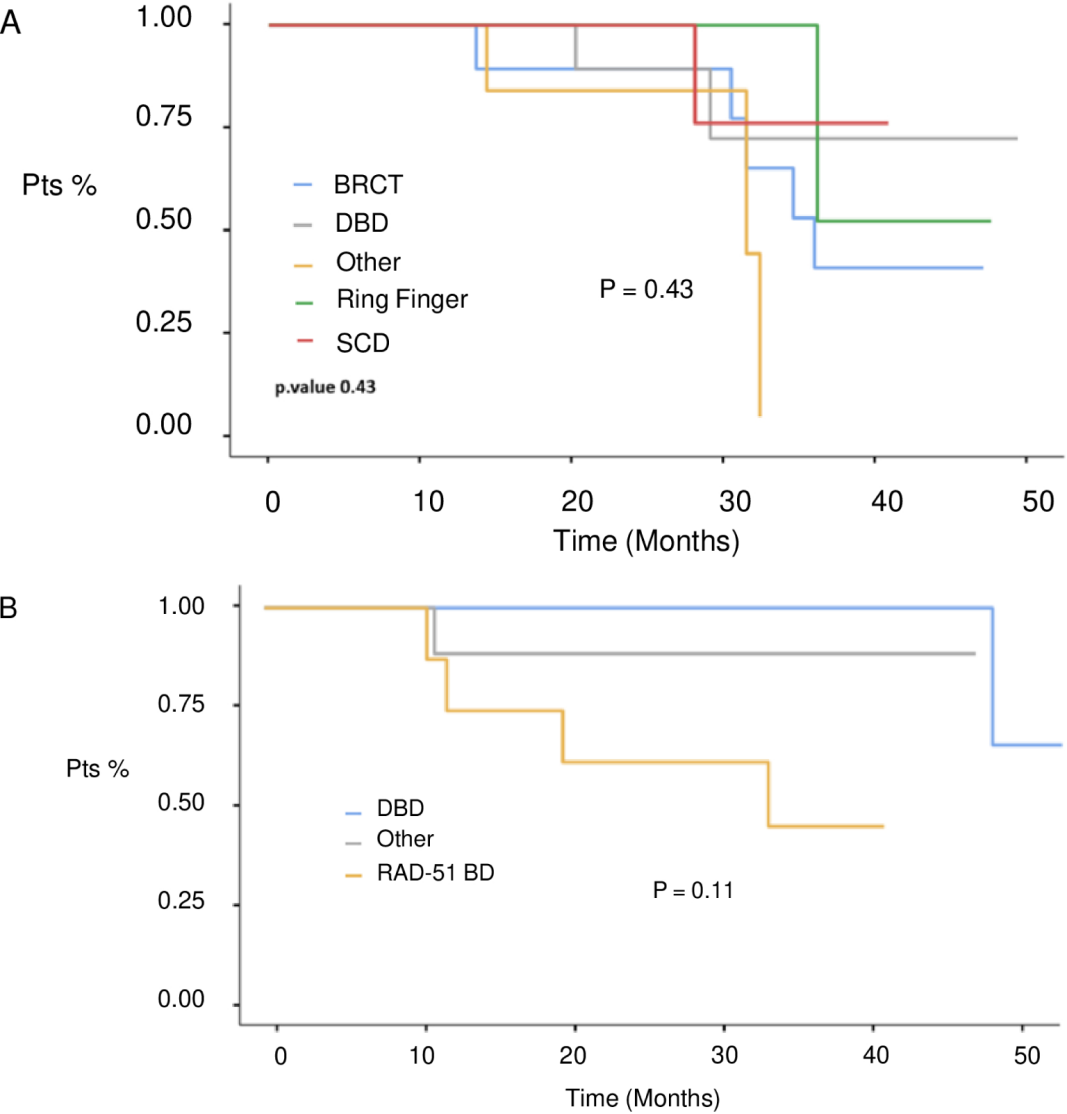
PFS in 1st line setting according to BRCA1 **(A)** and BRCA 2 **(B)** protein domains defects. BRCT, C-terminal domain of BRCA1; DBD, DNA-binding domain; RING, Really Interesting New Gene; RAD51 BD, RAD51-binding domain; SCD, serine cluster domain.

**Figure 4:**
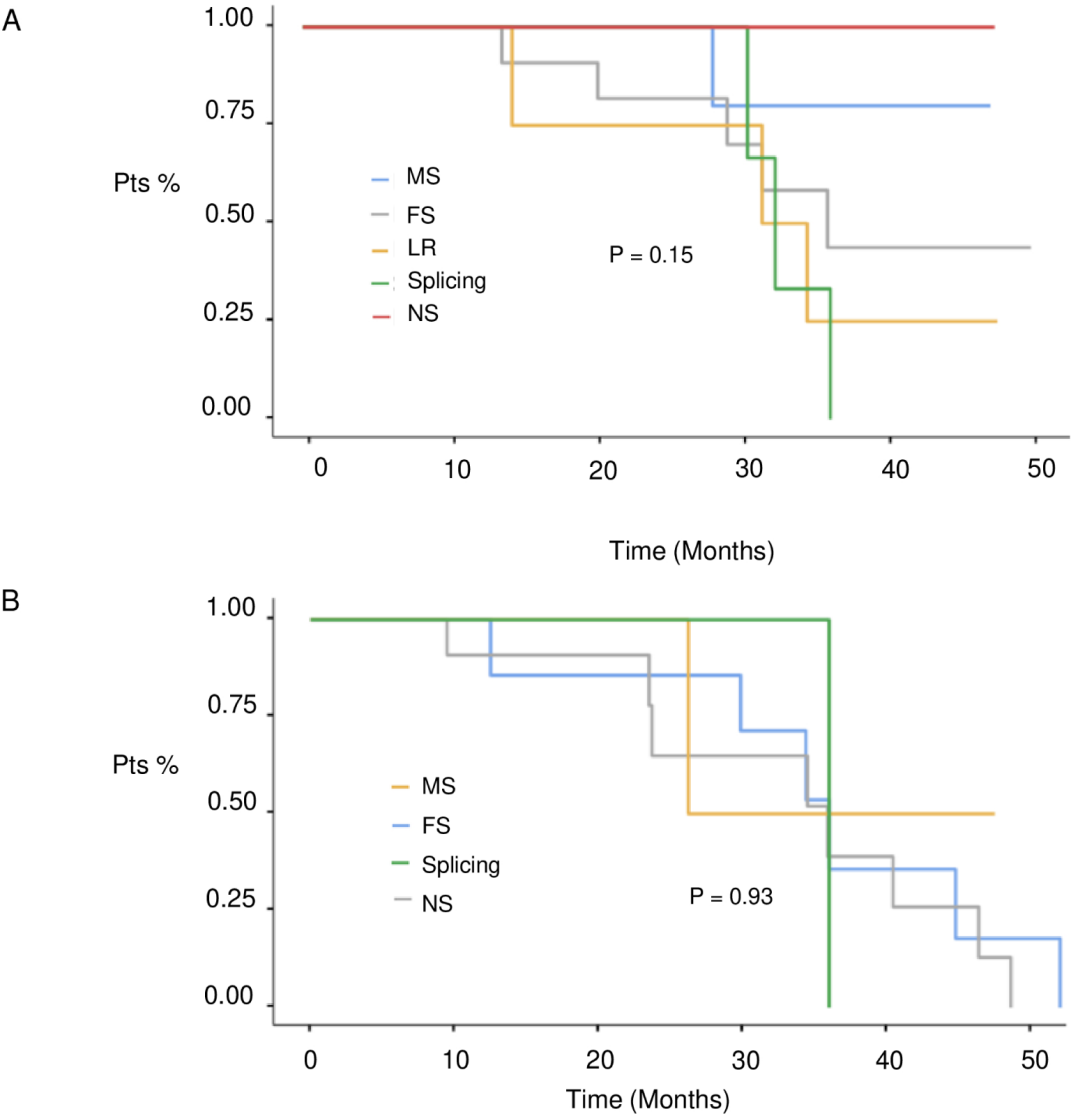
PFS in 1st line setting according to BRCA1 **(A)** and BRCA 2 **(B)** mutation type. MS, missense; FS, frameshift; MS, missense; LR, large rearrangement.

At a median follow-up of 51 months (IQR 17,4 months) no statistically significant difference in median PFS (mPFS) was observed between patients with BRCA1 or BRCA2 mutations who received first line platinum-based chemotherapy and PARPi (31.3 vs 30.1 months, respectively; hazard ration [HR] 0.77, 95% CI 0.29-2.07, p-value = 0.61) (**Figure 2**). Among patients with BRCA1-mutated HGSOC, those with defects in the DBD had the longest mPFS (33.8 months), while those with mutations in the Ring Finger Domain had the worst outcome (30.9 months). However, the magnitude of PARPi’s benefit across groups was not statistically significant (p-value = 0.43) (**Figure 3A**; **Table 2A**). In the BRCA2 cohort, patients with DBD mutations achieved a numerically longer, but not statistically significant, mPFS compared with those with mutations in RAD51-BD (39.8 months vs 24.1 months, p-value = 0.11) (**Figure 3B**; **Table 2A**). Additionally, PFS was analyzed according to the type of BRCA1/2 mutations. Among BRCA1 patients, those with LR mutations had the longest mPFS (33.2 months), while those with splicing mutations had the worst outcome (27.1 months; p-value = 0.15) (**Figure 4A**, **Table 2B**). Conversely, among BRCA2 patients, mPFS was longer in those with mutations in the MS domain (36.5 months), although no statistically significant correlation was observed (p-value = 0.93) (**Figure 4B**, **Table 2B**). A separate descriptive analysis was performed for patients treated with PARPi in the second-line setting. Results showed that patients with mutations in the BRCA1 Ring Finger Domain and BRCA2 DBD had the longest mPFS compared with other mutated protein domains. Furthermore, patients with FS mutations in BRCA1 and NS mutations in BRCA2 had improved outcomes based on mutation type classification. These findings were summarized in **Supplementary Tables 1A, B**.

**Table 2:** A. mPFS in 1st line setting in BRCA 1 and BRCA 2 subgroups according to protein domain.

	mPFS (months)	p-value
**BRCA 1**		0.43
Ring Finger Domain	30.9	
DNA Binding Domain	33.8	
Serine Cluster Domain	31.6	
BRCT Domain	32.2	
Other	26.1	
**BRCA 2**		0.11
RAD51 Binding Domain	24.1	
DNA Binding Domain	39.8	
Other	31.7	

**Table 2:** B. mPFS in 1st line setting in BRCA 1 and BRCA 2 subgroups according to mutation type.

	mPFS (months)	p-value
**BRCA 1**		0.15
Frameshift	31.7	
Large Rearrangement	32.1	
Nonsense	31.8	
Missense	31.5	
Splicing	27.7	
**BRCA 2**		0.93
Frameshift	31.4	
Nonsense	27.5	
Missense	36.5	
Splicing	35.7	

## Discussion

PARP inhibitors have gained a prominent position in the ovarian cancer treatment algorithm and have become a complementary tool to platinum agents. However, although PARP inhibition in the OC maintenance regimen has shown remarkable results, it is important to consider several prognostic and predictive factors, such as BRCA status, Homologous Recombination Deficiency, and residual disease, as they may affect the benefit observed from PARP inhibition. In our retrospective study, we aimed to explorethe potential impact of BRCA domain defects and type of BRCA mutation on PARP inhibitor efficacy. Our analysis showed that the benefit in terms of PFS from PARP inhibitors was observed regardless of the type and location of BRCA1/2 mutations. Our results align with recent findings from a *post-hoc* analysis of the phase III PAOLA-1 trial, which evaluated the effectiveness of adding olaparib or placebo to bevacizumab as first-line maintenance therapy in patients newly diagnosed with HGSOC and Homologous Recombination Deficiency. Among patients with a BRCA mutation, the combination of olaparib and bevacizumab was found to be highly effective, regardless of specific domain defects or the type of mutations (19). Although not statistically significant, our study also revealed a varying degree of benefit according to specific mutations. Specifically, among patients with *BRCA1* mutations, those with defects in the DBD achieved the highest PFS, while those with Ring Finger Domain mutations had the worst outcomes. These results align with previous preclinical and clinical evidence (19), which has suggested that RING domain-deficient BRCA1 may promote resistance to PARP inhibitors and platinum therapy in breast cancer cell lines harboring a hemizygous *BRCA1* 185delAG mutation (25). In particular, while initially sensitive to both PARPi and cisplatin, resistant clones emerged from native cells because of the increased expression of a RING domain deficient BRCA1 protein. Notably, this hypomorphic protein does not rely on the interaction with BAR D 1 for its activity, independently promoting RAD51 foci formation, DNA repair, and PARPi/platinum resistance (25). The potential impact of RING domain loss on PARPi activity has been further demonstrated in preclinical mouse models. In breast cancer chimeric mice carrying BRCA1185delAG and BRCA15382insC mutations, the activity of HRD targeted therapies, such as platinum agents and PARP inhibitors, has been evaluated. Intriguingly, mice expressing a BRCA1185delAG mutation, resulting in RING-less BRCA1 protein expression, developed PARPi resistance more rapidly than those harboring a BRCA15382insC mutation, suggesting that RING domain loss may serve as a potential marker of poor response to PARP inhibition (17). Conversely, consistent with our results, patients with defects in DBD enrolled in the PAOLA-1 trial were extremely sensitive to the olaparib plus bevacizumab combination (HR = 0.08; 95%CI [0.02, 0.28]) (19). In contrast, those randomized to the placebo group exhibited a poor response with a median PFS of 16 months compared to 19.9 months observed in patients with BRCT domains. Notably, the lack of benefit from platinum-based chemotherapy, which counteracts the impressive response to olaparib plus bevacizumab, may, at least in part, be related to the incomplete overlap between platinum and PARP resistance in these patients (19). Additionally, in the BRCA2 subgroup, patients harboring mutations in DBD achieved a median PFS longer than those displaying mutations in RAD51-BD. These results appear to be in line with preclinical evidence demonstrating a significant impact of DBD loss on cell survival and PARP inhibition (19). Specifically, clonogenic survival analyses upon treatment with DNA targeting agents such as olaparib, cisplatin, ionizing radiation, and mitomycin were conducted using mouse embryonic stem cells characterized by full length *BRCA2* gene or variants lacking DBD (ΔDBD), CTD (ΔCTD), DBD and CTD (ΔDBDΔCTD). Intriguingly, DBD loss led to heightened sensitivity to ionizing radiation and olaparib, regardless of CTD expression. Unexpectedly, DBD loss did not hinder the formation of RAD51 foci, BRCA2 diffusion, or immobilization. On the other hand, in the FACS-based BRCA2-targeting assay, BRCA domain defects prevented HR activation and affected BRCA2 architectural plasticity and its interaction with binding partners (18). Additionally, the DBD region has been reported to be less affected by the reversal of pathogenic BRCA mutations, in contrast to the high prevalence of those described in the N-terminal domain. It is worth mentioning that pathogenic BRCA mutations located in the DBD appear to be less prone to effective reversal due to the surrounding highly conserved sequences that play a central role in the HR (26).

Our study is one of the first real-world experiences to describe the potential impact of BRCA domain defects on PARP activity. Similar results have been reported by P. Torres-Mozas et al. in a retrospective study involving 26 HGSOC patients treated with either first-line (35%), first relapse (47%), or ≥second-line (18%) PARPi. In line with our findings, patients with RING domain mutations showed a poor response to treatment, whereas those with mutations in the DBD domain of *BRCA1* or *BRCA2* genes demonstrated a remarkable response (27). Furthermore, a larger retrospective analysis conducted by Lorusso et al. on 122 patients with *BRCA1* (60%) and *BRCA2* (40%) mutations showed that patients with DBD alterations had no survival events compared to those with other BRCA domain defects (log rank p = 0.079). Additionally, missense mutations were associated with longer progression-free survival (2 years PFS 100%) compared to splicing or nonsense mutations (log rank p = 0.021 and p = 0.049, respectively) (28). These results are in contrast with our observations and the results from the PAOLA-1 trial showing a reduced sensitivity, although not significant, to bevacizumab plus olaparib in patients with missense or splicing mutations. Additionally, the specific domain defects rather than the mutation type may have driven the benefit since no data regarding the distribution of missense mut in DBD domain were provided.

Our study has several limitations, including its retrospective nature and the small cohort of patients included in the analysis. The majority of patients in our study received Olaparib, rather than Rucaparib or Niraparib, limiting any reliable evaluation of potential differences between the PARPi. Furthermore, the number of patients treated with PARPi at relapse was too small to perform any statistical evaluation beyond a descriptive analysis. Additionally, full data on side effects and treatment adherence that may affect PARPi efficacy were not available.

## Conclusions

Our real-world dataset indicates that PARPi was effective regardless of BRCA specific domain defects or mutation type in patients with HGSOC who were treated with first-line platinum-based chemotherapy followed by PARPi maintenance. In terms of PFS, BRCA1 and BRCA2 DBD alterations were associated with the greatest benefits, while BRCA1 RING domain and BRCA1 splicing mutations showed the worst outcomes. This analysis provides intriguing evidence regarding the potential impact of BRCA specific domain defects on primary PARPi sensitivity. However, larger real-world datasets and collaborative studies are needed to further elucidate the impact of BRCA mut on predicting PARPi efficacy, in addition to platinum interval and HRD status.

## Data Availability

The raw data supporting the conclusions of this article will be made available by the authors, without undue reservation.
