# Supplementary material for: Efficacy of PARP inhibitors in advanced high-grade serous ovarian cancer according to BRCA domain mutations and mutation type
Source: Front Oncol. 2024 Sep 9;14:1412807. doi: 10.3389/fonc.2024.1412807 (PMC11416912; doi:10.3389/fonc.2024.1412807)
Supplement: Supplementary file 1 [file Table1.pdf]

| mPFS (months) | |
|---|---|
| **BRCA 1** | |
| Ring Finger Domain | 63.6 |
| DNA Binding Domain | 28.6 |
| BRCT Domain | 21.6 |
| Other | 64.8 |
| **BRCA 2** | |
| RAD51 Binding Domain | 22.1 |
| DNA Binding Domain | 49.6 |
| Other | 32.5 |

**Supplementary Table 1A. mPFS in 2nd line setting in BRCA 1 and BRCA 2 subgroups according to protein domain.**

| mPFS (months) | |
|---|---|
| **BRCA 1** | |
| Frameshift | 45.4 |
| Large Rearrangement | 28.3 |
| Missense | 35.2 |
| Nonsense | 32.8 |
| **BRCA 2** | |
| Frameshift | 16.5 |
| Splicing | 6.7 |
| Missense | 23.6 |
| Nonsense | 64.9 |

**Supplementary Table 1B. mPFS in 2nd line setting in BRCA 1 and BRCA 2 subgroups according to mutation type.**
